# SuiteMSA: visual tools for multiple sequence alignment comparison and molecular sequence simulation

**DOI:** 10.1186/1471-2105-12-184

**Published:** 2011-05-21

**Authors:** Catherine L Anderson, Cory L Strope, Etsuko N Moriyama

**Affiliations:** 1 Department of Computer Science and Engineering, University of Nebraska, Lincoln, Nebraska 68588, USA; 2 School of Biological Sciences, University of Nebraska, Lincoln, Nebraska 68588, USA; 3 Center for Plant Science Innovation, University of Nebraska, Lincoln, Nebraska 68588, USA

## Abstract

**Background:**

Multiple sequence alignment (MSA) plays a central role in nearly all bioinformatics and molecular evolutionary applications. MSA reconstruction is thus one of the most heavily scrutinized bioinformatics fields. Evaluating the quality of MSA reconstruction is often hindered by the lack of good reference MSAs. The use of sequence evolution simulation can provide such reference MSAs. Furthermore, none of the MSA viewing/editing programs currently available allows the user to make direct comparisons between two or more MSAs. Considering the importance of MSA quality in a wide range of research, it is desirable if MSA assessment can be performed more easily.

**Results:**

We have developed SuiteMSA, a java-based application that provides unique MSA viewers. Users can directly compare multiple MSAs and evaluate where the MSAs agree (are consistent) or disagree (are inconsistent). Several alignment statistics are provided to assist such comparisons. SuiteMSA also includes a graphical phylogeny editor/viewer as well as a graphical user interface for a sequence evolution simulator that can be used to construct reference MSAs.

**Conclusions:**

SuiteMSA provides researchers easy access to a sequence evolution simulator, reference alignments generated by the simulator, and a series of tools to evaluate the performance of the MSA reconstruction programs. It will help us improve the quality of MSAs, often the most important first steps of bioinformatics and other biological research.

## Background

Multiple sequence alignment (MSA) plays a central role in nearly all bioinformatics and molecular evolutionary applications. Be it to discover sequence structure and motifs or to infer the evolutionary history among sequences (phylogeny), the first step is to compare the sequences by building MSAs. The process of building an MSA is to infer homologous positions between the input sequences and place gaps in the sequence in order to align these homologous positions. These gaps represent evolutionary events of their own. Gaps (also called indels) are caused by either insertions or deletions of characters (nucleotides or amino acids) on a particular lineage of sequences during the evolution. In this sense, building an MSA is to reconstruct the evolutionary history of the sequences involved.

Due to its significant impact on many bioinformatics and molecular evolutionary analyses, MSA reconstruction is one of the most heavily scrutinized bioinformatics fields. Numerous MSA reconstruction methods have been developed [1]. Assessment of MSAs, however, is usually reserved for power users. Often regular users simply run one MSA method and proceed directly to the next analysis without examining the alignment output. Considering the importance of MSAs, it is desirable if quality assessment of MSA methods can be performed more easily and more intuitively by all researchers who are interested in sequence analysis. There are a number of programs available that generate, display, and/or let users analyze MSAs such as SeaView [2], ClustalX2 [3], Se-Al [4], Jalview [5], webPRANK [6], as well as MEGA [7]. However, none of these programs allows the user to make direct comparisons between two or more MSAs. SinicView [8] can visualize multiple MSAs. Its use, however, is targeted for genome-scale nucleotide alignments, and position-by-position comparison among MSAs is not possible. As Morrison [9,10] also pointed out, visual inspection of multiple MSAs would greatly help improve the quality of MSAs and consequently the reconstruction of phylogenies.

Effective evaluation of MSA methods requires reference alignments. These are the MSAs that are considered to represent the evolutionary history of the sequences most accurately. The majority of currently available benchmark MSA datasets are based on structural alignments of real sequences (*e.g*., PREFAB [11], OXBench [12], HOMSTRAD [13], BAliBASE [14], SABmark [15], also see Edgar [16] for some issues with these benchmark datasets) where the actual evolutionary history is unknown. Researchers, especially those very familiar with their sequences, often adjust MSAs manually. This introduces several issues. There is no "standard" way to adjust/improve an alignment. It is very time consuming and alignments often cannot be fully resolved. A solution to these issues is offered by Hillis [17]. He pointed to sequence evolution simulation as an alternative method to obtain reference MSAs and analyze MSA algorithms. Sequence evolution simulation methods generate a set of related nucleotide or amino acid sequences with a known evolutionary history, *i.e*., providing a fully-resolved MSA. The datasets generated by simulation, with various evolutionary parameter settings, are also useful for evaluating the robustness, consistency, and efficiency of phylogenetic reconstruction based on different MSA methods. The disadvantage of using simulated sequences, however, is that the events during the simulated evolution are limited by the evolutionary models available in the simulators. One must thus choose an appropriate simulator that can mimic the evolutionary history of the gene or protein sequences he/she is interested in.

Many molecular evolution simulation programs are currently available: *e.g*., INDELible [18], Rose [19], DAWG [20], MySSP [21], SIMPROT [22], EvolveAGene3 [23], and indel-Seq-Gen version 2.1 (iSGv2.1) [24]. Rose [19] has been used to generate IRMBASE 2 and DIRMBASE benchmark alignment datasets [25]. All of these programs require several input files and run on the command line. One exception is MySSP, which is run from a simple graphical user interface (GUI). Of the available simulation programs, iSGv2.1 is the most versatile and complex. It allows for subsequences or sites to evolve with less stringent assumptions, *i.e*., relaxing the assumption of the independent-and-identically-distributed sequence sites, which is prevalent in the field of molecular evolution simulation. iSGv2.1 thus can generate more realistic protein and gene families [24]. Such complex and more realistic simulation, however, requires detailed input files and numerous options with the command line.

We introduce SuiteMSA, a suite of graphical tools for MSA comparison that also encapsulates the sequence evolution simulation program, iSGv2.1. SuiteMSA offers tools that allow for the direct comparison of multiple MSAs. These tools assist researchers to visually pinpoint the areas where alternative MSAs are inconsistent with a reference MSA, which can be either an MSA obtained from a benchmark MSA database, a manually curated MSA, or a true MSA based on simulated sequences. Statistics to aid the quantitative comparisons of MSAs are provided. SuiteMSA also allows users of any level to perform simulation of biological sequence evolution. With intuitive option panels users can quickly set up an evolutionary model for simulation. After the simulation, SuiteMSA displays and maps indel events to the true MSA and also to the simulation guide tree. This immediate feedback is useful in inspecting the simulated datasets, allowing the user to choose the set of simulation parameters that is best able to produce datasets with the desired features. Providing sequence simulation as well as MSA assessment capability is educational in understanding how various MSA methods work differently when biological sequences have different evolutionary properties.

## Implementation

**Figure 1:**
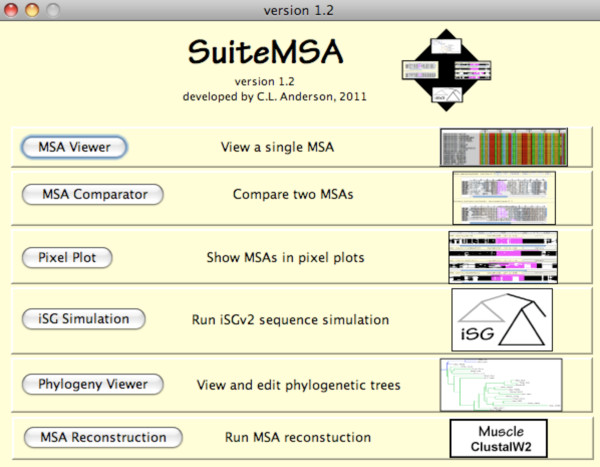
**The main window of SuiteMSA**. The main window shows the six main tools of SuiteMSA. Clicking on each button brings up the specific tool.

SuiteMSA is a java-based application that provides unique MSA viewers (Figure 1). It can be used with any MSA in fasta format. Individual alignments may be viewed along with secondary structure (both for proteins and RNAs) or transmembrane predictions. Users can directly compare multiple MSAs and evaluate where the MSAs agree (are consistent) or disagree (are inconsistent) visually as well as quantitatively based on several statistics. SuiteMSA also allows visual inspection and editing of phylogenies. Furthermore, it provides a GUI for a sequence simulator, iSGv2.1. Once communication is set up between SuiteMSA and iSGv2.1, parameters can be configured and simulations can be launched from SuiteMSA. A log tracks all simulations performed recording the information including parameters used, date and time stamps, error messages, and all communications with iSGv2.1. Once the simulation is done, the true MSA and phylogeny with indel events mapped can be displayed. Note that MSA viewers in SuiteMSA are independent of the simulation program and do not require the installation of iSGv2.1.

### A case study: the lipocalin protein superfamily

**Figure 2:**
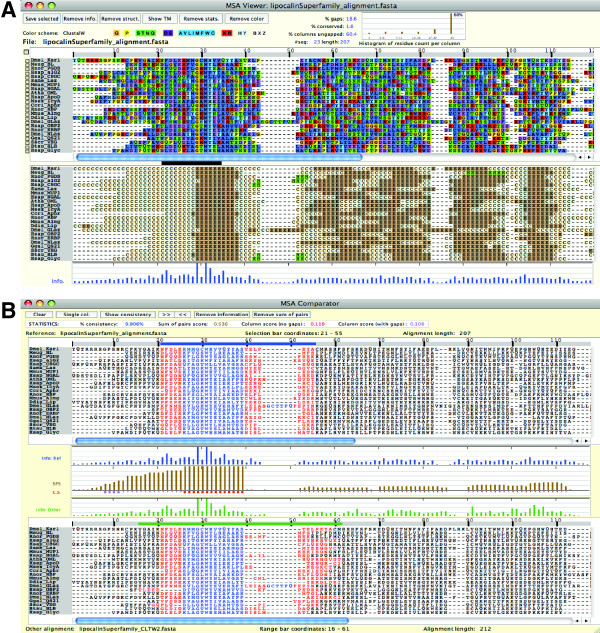
**The MSA Viewer and MSA Comparator**. The reference alignment of the lipocalin superfamily proteins is displayed using the MSA Viewer (A). The secondary structures predicted by PSIPRED [29] are shown below the alignment. The information content [43] of each column of the alignment is displayed at the bottom, which illustrates the level of conservation. The completely conserved positions in the motif (positions 29 and 31; the motif region is indicated with the black bar) show the maximum information content (full-height bars). In the MSA Comparator (B), the ClustalW2 alignment (shown as "Other alignment") is compared against the "Reference" alignment. The blue selection bar, which is set to cover 35 sites, is shown above the reference alignment. The green range bar above the ClustalW2 alignment shows the column range that covers the characters selected in the reference alignment. The colored characters under the selection and range bars in the alignments show the sequence positions either in agreement (consistent) in blue, or else in red. The consistent (blue) columns largely correspond to the conserved motif (PROSITE motif PS00213). The column-wise Sum-of-Pairs Score (SPS) is shown between the two alignments. The SPS, column scores including and excluding gap columns ("with gaps" and "no gaps", respectively), and percent consistency for the alignment are shown in the statistics bar at the top of the window.

We use multiple alignments and simulation of the lipocalin superfamily proteins as a case study to illustrate how the MSA comparison and simulation can be done with SuiteMSA. Lipocalin proteins are a family of small globular proteins often implicated in allergic reactions, among other functions. Members of the lipocalin superfamily have low sequence identity, but share a common antiparallel beta-barrel conformation consisting of eight beta-strands, as well as a small highly-conserved motif near the first beta-strand [26]. We obtained both the manually-adjusted MSA and phylogeny reconstructed from 23 members of the lipocalin superfamily from Sánchez *et al*. [27]. Strope *et al*. [28] used the same example to introduce iSGv1. We will use this MSA and guide tree in this case study. Figure 2A shows this MSA using the *MSA Viewer *along with the display of the secondary structures predicted by PSIPRED [29] for each sequence. The 23 lipocalin protein sequences, the alignment and phylogeny, and the predicted secondary structures are available in Additional files 1, 2, 3, and 4.

### MSA comparison

**Figure 3:**
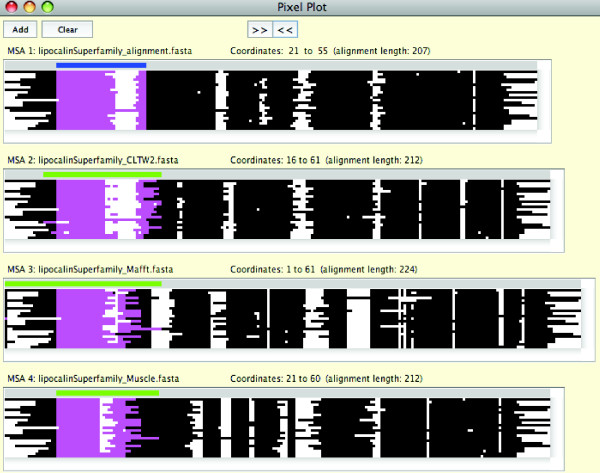
**The Pixel Plot**. The Pixel Plot is used to compare the reference alignment of the lipocalin superfamily proteins (MSA 1) with MSAs reconstructed by three methods: ClustalW2 v2.1 (MSA 2), MAFFT v6.843 (MSA 3), and MUSCLE v3.8.31 (MSA 4). The blue selection bar is shown at the top of the reference alignment. The green range bar above each of the reconstructed alignments shows the column range that covers the sequence positions selected in the reference alignment. The areas highlighted in magenta under the selection and range bars in the alignments show the positions of the characters selected in the reference alignment.

SuiteMSA offers two unique tools to compare MSAs: *MSA Comparator *(Figure 2B) and *Pixel Plot *(Figure 3). For both tools, MSAs are compared against the reference MSA (at the top). The blue and green bars shown above MSAs in Figures 2B and 3 are the "selection bar" for the reference MSA and the "range bar" for the alternative MSAs, respectively. The selection bar indicates the selected region in the reference MSA. The range bar for an alternative MSA indicates the range covered by the characters selected in the reference MSA. This makes the MSA comparison easy, visually depicting the difference in alignment extension/compaction or gappiness.

The *MSA Comparator *allows the user to perform a fine-grained comparison between two alignments. Figure 2B compares the reference MSA of the lipocalin superfamily proteins (shown also in Figure 2A using the single alignment viewer, *MSA Viewer*) with the alignment reconstructed by ClustalW2 v2.1 [3]. Alignment positions under the selection and range bars are color-coded for consistency with respect to the reference MSA. Characters in consistently aligned columns are colored blue, and those in columns inconsistently aligned are colored red. In Figure 2B, for example, the highly conserved area surrounding the position 29 of the reference alignment is consistent between the two MSAs and colored blue, whereas after the position 40 the MSAs are inconsistent and so colored in red. The column-wise Sum-of-Pairs Score (SPS) [30] is also displayed using a bar chart in Figure 2B, with maximum-height bars shown for consistent columns (positions 26 - 39). The SPS shows the degree of consistency per column between the two alignments. For detailed description of these measures see SuiteMSA user's manual.

The *Pixel Plot *allows for a quick comparison between multiple MSAs. As shown in Figure 3, each character in the MSA is represented as a solid colored pixel and each gap as a blank pixel. In Figure 3, the reference alignment of the lipocalin superfamily proteins (at the top) is compared with the three MSAs reconstructed by ClustalW2 v2.1 [3], MAFFT v6.843 [31], and MUSCLE v3.8.31 [32]. The selected characters for the reference alignment (MSA 1) under the blue selection bar and the corresponding characters for the reconstructed alignments (MSAs 2-4) under the green range bars are colored in magenta. This is the same area as selected in Figure 2B.

### Sequence simulation

Simulating members of the lipocalin protein superfamily represents a challenge for many simulators because (i) due to the short length of the lipocalin proteins, each of the 19 subsequences (eight beta-strands, one alpha-helix, and ten coil regions) has a strict length constraint and (ii) all members of the family must contain the conserved motif (PROSITE PS00213 [33,34]) near the first beta-strand. In this section, we set up options for iSGv2.1 for the lipocalin family simulation. The parameters were chosen by the following procedure:

• The phylogeny reconstructed by Sánchez *et al*. [27] was used as the simulation guide tree.

• The alignment presented in Sánchez *et al*. [27] was used as the root MSA.

• We analyzed Sánchez *et al*.'s alignment using the PROTTEST Web server [35-38] using the guide tree topology. The model that best fit the data was the WAG substitution matrix [39] with the Gamma distribution (alpha = 3.88). The amino acid frequencies as well as the branch lengths for the phylogeny were also estimated by PROTTEST.

• We estimated the indel parameters based on the reference alignment and guide tree using the lambda.pl program from the DAWG package [20]. The geometric distribution with the average length of 6.97 as the length distribution model and the indel probability of 0.0516702 per substitution were returned. We assumed the maximum length of an indel to be 20 amino acids.

**Figure 4:**
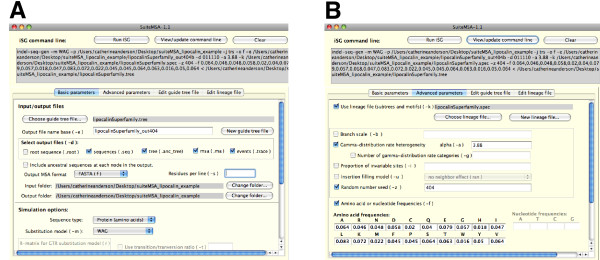
**Setting simulation parameters**. Parameters for the iSGv2.1 simulation are set in the "Basic parameters" panel (A) and in the "Advanced parameters" panel (B). In the "Basic parameters" panel, the guide tree file is set to "lipocalinSuperFamily.tree". The file format, output file name prefix, output files, as well as the sequence type and substitution model are set in this page. In the "Advanced parameters" page, the lineage specification file, which contains the motifs to be applied to the simulation, is selected. The alpha constant for the Gamma distribution is set to 3.88 and the amino acid frequencies are set as shown in this example.

SuiteMSA makes setting up the iSGv2.1 simulation with all these options easily accessible through the GUI. Parameters and support files are organized into four panels based on their intuitive groupings: "Basic parameters", "Advanced parameters", "Edit guide tree file", and "Edit lineage file". Figure 4A shows how we can set the basic simulation parameters including the input/output file names and substitution models. Figure 4B shows the advanced simulation parameters including the alpha constant for the Gamma distribution and amino acid frequencies.

**Figure 5:**
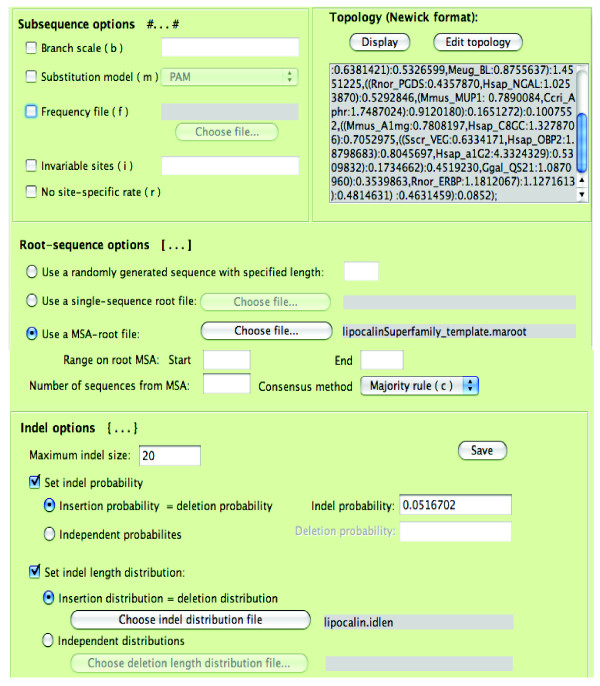
**The "Edit guide tree file" panel**. The guide tree file used by iSGv2.1 contains the partition-specific parameters. Using the "Edit guide tree file" panel users can set all necessary parameters including the guide tree. In this example, the MSA-root file for the lipocalin superfamily contains the alignment obtained from Sánchez *et al*. [27]. Indel options are also set as shown. The guide tree can be edited either by editing the tree in Newick format or by using an interactive graphical display (see Figure 6).

**Figure 6:**
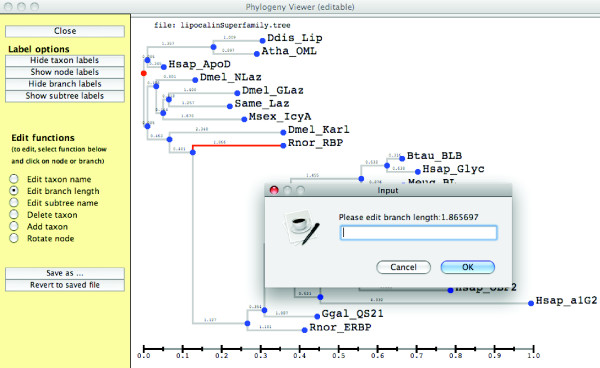
**Editing the guide tree graphically**. The graphical tree editor allows the user to change taxon names, change branch lengths, delete or add taxa, rotate clades, and add clade names. In this example, a branch is chosen for its length to be changed.

The simulation guide tree must be provided in a guide tree file in Newick format. The guide tree file also specifies other parameters including indel parameters. These additional parameters can be set in the "Edit guide tree file" panel (Figure 5). SuiteMSA also allows the input guide tree to be modified using a graphic tree editor (Figure 6). Editing the guide tree graphically, such as changing the taxon labels, adding clade names, changing branch lengths, adding/deleting taxa, or rotating clades, provides immediate feedback ensuring the accuracy of the changes made.

**Figure 7:**
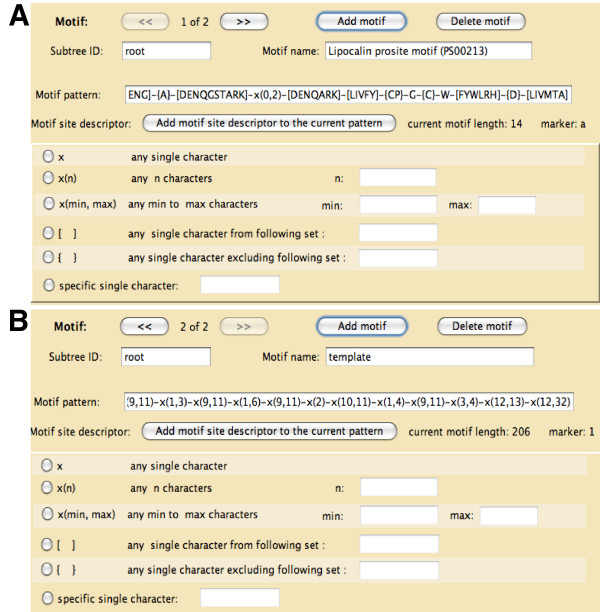
**Editing the lineage specification file**. The lineage specification file used by iSGv2.1 allows the users to specify motifs (A) and templates (B) for different lineages (subtrees or clades). In this example, the motif "Lipocalin prosite motif (PS00213)" is set for the "root" lineage, which covers the entire guide tree. Another motif "template" is used to provide the length constraints for 19 subsequences.

In the "Edit lineage file" panel, lineages (clades or subtrees) can be added or deleted, and lineage specifications can be edited. In the panel shown in Figure 7A, we can edit or add motif specifications through a regular expression generator. Figure 7B illustrates a special use of this regular expression generator to specify the subsequence-length "template". The example includes constraints for 19 subsequences. See Strope *et al*. [24] for the details of the use of templates. All support files used for this example simulation are available in Additional files 5, 6, 7, and 8.

Prior to running a simulation, SuiteMSA provides error-checking for potential parameter conflicts. The actual command line used to run iSGv2.1 with all necessary options is shown at the top of the *iSG Simulator *window as illustrated in Figure 4. The simulation log file saves the parameters used along with any messages from iSGv2.1. This log can also be useful for retrieving the saved iSGv2.1 command-line for a later use.

**Figure 8:**
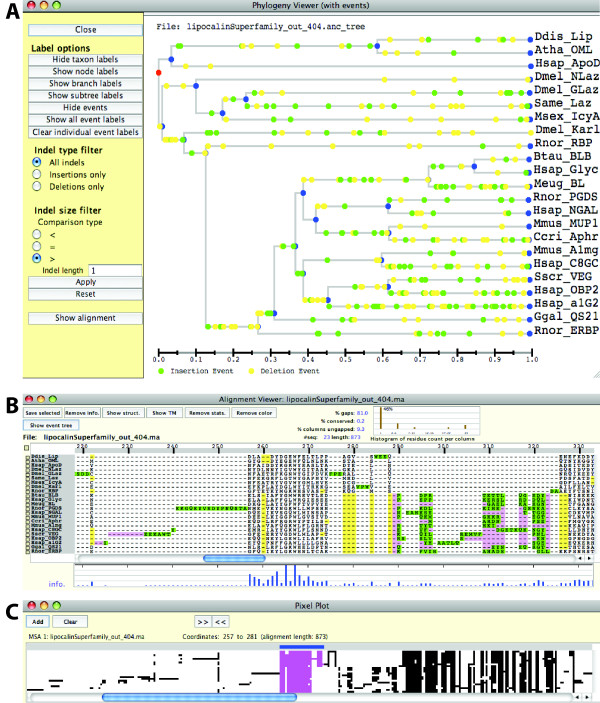
**The display tools for the simulated datasets**. The Phylogeny Viewer (A) shows indel events on the guide tree in a time relative manner. Filters can be applied to show only a specific type of events (insertions or deletions). The Alignment Viewer (B) shows the alignment with indel events color-coded. Insertions are shown in green, deletions in yellow, and if both events happen in the same position, in pink. The block that has no gap, from positions 257 to 274, contains the PROSITE motif. The information content bar chart beneath the alignment illustrates that the positions 265 and 267 are completely conserved. The Pixel Plot (C) visualizes a larger portion of the alignment, illustrating the general pattern of the alignment.

After a simulation is done, insertion and deletion events can be tracked on the guide tree using the *Phylogeny Viewer *(Figure 8A) and in the true MSA using the *Alignment Viewer *(Figure 8B). In these viewers, insertion and deletion events are shown in different colors: insertion events in green and deletion events in yellow. In the *Alignment Viewer*, where an insertion and a deletion event occur in the same location, the site is shown in pink (*e.g*., positions 226 - 233 in Figure 8B). These viewers can display any phylogeny and MSA in the appropriate format (Newick format for phylogenies and fasta format for MSAs). Note, however, that indel-event mapping is only available when phylogenies or MSAs are associated with iSGv2.1 simulation. Figure 8C illustrates the use of the *Pixel Plot*, which displays a wider region of the same alignment with the blue selection bar indicating the positions 257 - 281.

### Graphical interface for MSA methods

**Figure 9:**
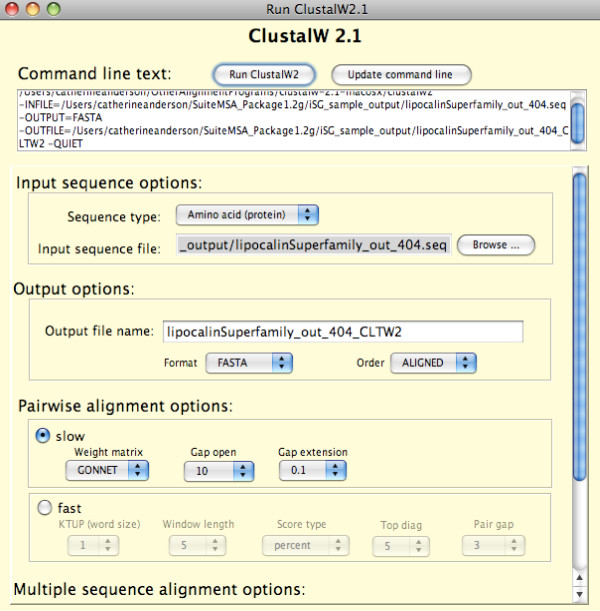
**The graphical user interface for ClustalW2**. SuiteMSA provides a GUI for ClustalW2. The resulting alignments can be displayed using the MSA Viewer, MSA Comparator, or Pixel Plot.

To assist comparative studies of MSAs, SuiteMSA offers GUIs for some MSA programs (Figure 9). To use this function, the appropriate MSA programs need to be installed. Once installed, the MSA programs can be run and the resulted alignment viewed through SuiteMSA. Currently, GUIs for ClustalW2 [3,40] and MUSCLE [32,41] are available. We plan to expand our support for other commonly used MSA methods (*e.g*., MAFFT [31]) in the future.

## Results and Discussion

As we described before, the performance of MSA methods can be examined against a reference MSA. A reference MSA can be obtained from a benchmark MSA database or by manually-adjusting any MSA relying on our own experience and knowledge on the sequences of our interests. Or we can use a sequence simulator that generates a "true" MSA based on the given evolutionary model. In the previous section, we used the lipocalin superfamily proteins as a case study, and showed how we can simulate members of such a complex protein family. Simulated protein sequences were aligned using different MSA methods. In this section, as a further example, we will briefly discuss how these reconstructed MSAs are compared with the "true" MSA obtained from the simulation as well as the manually adjusted alignment produced by Sánchez *et al*. [27].

In Figure 2B, Sánchez *et al*.'s alignment of lipocalin proteins is used as the reference alignment (at the top) and compared with the alignment reconstructed by ClustalW2. In this alignment, the area containing the PROSITE motif (positions 21 through 34 in the reference alignment) is mostly colored blue showing a high degree of consistency. Note that the first five positions of the motif are not consistent (shown in red) due to the gaps inserted in the ClustalW2 MSA. The entire motif region, however, maintains high SPS values. However, the characters in the positions 40 through 44 in the reference alignment are scattered over 17 columns in the ClustalW2 MSA and colored in red, with 0 or very low SPS values. We expanded the comparison and included MSAs reconstructed by MUSCLE and MAFFT using the *Pixel Plot*. As shown in Figure 3, the three methods show their MSAs (MSAs 2-4) consistent with the reference MSA (MSA 1) at the left edge of the conserved motif region, indicated by the nearly straight edge marked in magenta in all alignments. However, there is a high degree of inconsistency in the downstream section between the reference alignment and reconstructed MSAs and even among the three reconstructed MSAs.

**Figure 10:**
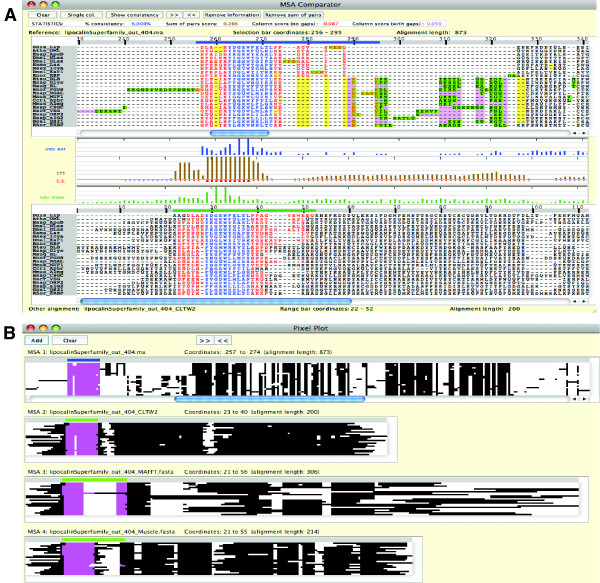
**MSA comparisons for simulated lipocalin-superfamily protein sequences**. Twenty-three protein sequences of the lipocalin superfamily were simulated using iSGv2.1. Simulated sequences were aligned using ClustalW2 v2.1, MAFFT v6.846, and MUSCLE v3.8.31. Panel A shows the comparison of the MSA reconstructed by ClustalW2 against the true alignment provided by iSGv2.1 (the reference alignment) using the MSA Comparator. The column-wise SPS along with the information content for each alignment are displayed between the two alignments,. In Panel B, the Pixel Plot is used to compare the three reconstructed MSAs (MSAs 2-4) against the reference alignment (MSA 1).

Comparison against the true alignment based on simulated lipocalin protein sequences showed the same pattern (Figure 10): a high degree of consistency in the area where the PROSITE motif is located (sites 258 - 270 of the reference MSA) and a high degree of inconsistency on the right-hand side of this area. In this reference alignment, we see many indel events immediately preceding the PROSITE motif (the position 257 or upstream). As Figures 8B and 8C show, this area includes several insertions followed by deletions at the same sites, resulting in gap-only columns in the reference alignment (colored in pink in Figure 8B). As shown in Figure 10B, this area (~250 amino-acid sites in the reference) has been compressed into 25 columns in all three reconstructed MSAs (MSAs 2-4). While many indel events are found in the region following the PROSITE motif (downstream of the position 270) in the reference alignment, this region is again compressed in all three reconstructed MSAs, although less so in the MAFFT MSA. Regardless of the lengths and gappiness, this area has been poorly reconstructed by the three MSA programs. As a consequence of compressed alignments, the three reconstructed MSAs are much shorter than the reference MSA (Figure 10B; alignment lengths are 873, 200, 306, and 214 amino acids for MSAs 1-4, respectively). Löytynoja and Goldman [42] pointed out that progressive alignment algorithms, used in all of ClustalW2, MUSCLE, and MAFFT, tend to produce compacted alignments due to "collapsed insertion" and "gap magnet" problems. The results shown in Figure 10B indicate that such compaction is particularly pronounced in ClustalW2 and MUSCLE MSAs. Further investigation is clearly necessary in order to choose better MSAs.

## Conclusions

SuiteMSA provides unique MSA viewers, which allow researchers to quickly identify inconsistencies among MSAs reconstructed by different techniques. It assists in performance evaluation of MSA methods. SuiteMSA also allows users to perform sequence simulation. This further assists comparative analysis of MSAs based on the "true" reference alignment where insertion and deletion events can be mapped individually onto both the guide tree and the true MSA. SuiteMSA's intuitive and user friendly GUI allows for a quick learning curve in using the powerful simulation program iSGv2.1. This provides an opportunity to a wide range of researchers for setting up complex simulation studies quickly and accurately. With the MSA Viewer, MSA Comparator, Pixel Plot, as well as a graphical sequence simulator, the Phylogeny Viewer with graphical editing options, and the Alignment Viewer with indel-event tracking, SuiteMSA contributes a wide variety of unique features to the field of multiple sequence alignment, sequence evolution simulation, and more general bioinformatics research.

## Availability and requirements

• Project name: SuiteMSA

• Project home page: http://bioinfolab.unl.edu/~canderson/SuiteMSA/

• Operating system(s): Mac OS X 10.5 or higher, Linux, and Unix

• Programming language: java 1.6

• Other requirements: iSGv2.1 must be installed per instructions for sequence simulation. ClustalW2 and MUSCLE need to be installed if the user wish to use the GUIs provided with SuiteMSA.

• License: none

• Any restrictions to use by non-academics: none

## Authors' contributions

CLA implemented SuiteMSA and wrote the manuscript. CLA, CLS, and ENM conceived the project, discussed the results, and revised the manuscript. All authors have read and approved final manuscript.

## Supplementary Material

Additional file 1 **23 lipocalin protein sequences**. The 23 lipocalin protein sequences obtained from Sánchez *et al*. [27] are included in fasta format. Click here for file

Additional file 2 **The reference alignment of 23 lipocalin proteins**. The alignment of the 23 lipocalin protein sequences obtained from Sánchez *et al*. [27] is included in fasta format. Click here for file

Additional file 3 **The phylogenetic tree of 23 lipocalin proteins**. The phylogenetic tree of the 23 lipocalin protein sequences obtained from Sánchez *et al*. [27] is included in Newick format. Click here for file

Additional file 4 **Secondary structure predictions for 23 lipocalin proteins**. The secondary structures for the 23 lipocalin proteins predicted by PSIPRED are provided in fasta format. Click here for file

Additional file 5 **The guide tree file**. File name: lipocalinSuperfamily.tree. This file contains the guide tree along with other parameters used for the iSGv2.1 simulation of the lipocalin superfamily proteins. In addition to this file, Additional files 6-8 are used for the simulation. A brief instruction for setting up SuiteMSA using these files and running the simulation as described in the case study is included in the SuiteMSA package distribution. Click here for file

Additional file 6 **The simulation root MSA**. File name: lipocalinSuperfamily_template.maroot. This file contains the multiple sequence alignment along with the patterns for the motif and template used for the lipocalin superfamily simulation. Click here for file

Additional file 7 **The lineage specification file**. File name: lipocalinSuperfamily.spec. This file contains the motif specifications for lineages used for the lipocalin superfamily simulation. Click here for file

Additional file 8 **The indel-length distribution file**. File name: lipocalin.idlen. This file contains the indel-length distribution used for the lipocalin superfamily simulation. Click here for file
